# 

**Published:** 1912-08-31

**Authors:** 


### Buildings Contemplated.

Barham (Suffolk).—Tenders are under consideration of the Bosmere and Claydon Rural District Council for an observation block at the Barham Infectious Hospital.

Christchurch.—The Christchurch Guardians are making application for authority to provide a new laundry to cost £3,363, and to convert the existing laundry into stores at a cost of £544.

Debreczen (Hungary).—The *Zentral-Anzeiger* (Vienna) announces that the municipal authorities of Debreczen are negotiating a loan, including a sum of £125,000, for the erection of a hospital.

Derby.—Subject to a grant under the Insurance Act it is proposed to establish a tuberculosis dispensary, enlarge the existing sanatorium, and arrange for establishing ten or twelve beds for cases requiring prolonged treatment.

### BUREAU OF INFORMATION.

### Rules for Correspondents.

1. Every letter, on whatever subject, must be accompanied by the coupon to be cut from the back cover (inside page) of The Hospital, current issue, and must contain the name and address of the correspondent with pseudonym for publication if desired.
2. Replies by post cannot be given, save under exceptional circumstances at the Editor's discretion.
3. Letters from Approved Homes in reply to special needs published in the Bureau should state terms and full particulars, and be sent prepaid under cover to the Editor of the Bureau with coupon and name enclosed for identification.
4. Proprietors of Homes which have not yet been entered on the List of Approved Homes, but have spare accommodation likely to suit special needs, are invited to write for an application form for registration. The fee for registration, which includes two announcements of the Home in the Bureau, is 5s.
5. Special needs of every description relating to those who are sick or in difficulties are made known in these columns free of charge.
6. All communications to be addressed to The Editor of The Hospital, 28 Southampton Street, Strand, London, W.C., and marked "Bureau of Information."

### Needs and Helpers.

### Aid in First Confinement.

Home Wanted.—We have much sympathy with you and will give you every help in our power. Write, giving full particulars, to the Sister Superior, St. Mary Magdalene Maternity and Convalescent Home, Paddington. We have already mentioned the case to her, and feel sure your friend will be happy and usefully employed while in residence, with all anxieties removed and a prospect of future good employment. Let us hear again.

### Home for Step-child.

A. K. C.—There is every hope for your young parishioner if she is placed under skilled direction with a wise blend of love and discipline. We recommend you to write to the Sister-in-Charge, St. Helena's Home, Ealing. The girls there are very happy and do remarkably well, though there is invariably a past record of insubordination and misconduct. It is not a rescue home. The charge is small.

### Home for Chronic Invalids.

Lover of Justice.—Your delightful letter has given us great pleasure. It is admirable to find you all writing to give testimony to the excellence of the Alexandra Home, Wimbledon, and we shall not forget that it affords "good food and plenty of it, four meals a day, a comfortable ward and bed, and attendance, and in winter good fires." But in our judgment the sweet fragrance of grateful hearts outweighs even these comforts. Long may it prosper!

### Blind and Deaf.

D. B. (Maidstone).—We admire your energy and have no doubt it will be rewarded in the end. We can give you more addresses if by ill-luck you do not succeed with the society you name. Remember "it is dogged that does it."

### Training and Employment.

### Training for Girl of Nineteen.

A. M. G. (Newlands).—The Norland Institute is at 10 Pembridge Square, London, W. The training includes three months' experience in a children's hospital. Princess Christian's College is at 19 Wilmslow Road, Manchester. There is also the Sesame House, 43a Acacia Road, St. John's Wood, London, N.W. In none of these colleges, however, are pupils accepted without a premium of from £30 to £50. Your daughter will not be eligible for training in any good fever hospital until she is twenty. We advise you to write to the Hon. Secretary, The Fever Nurses' Association, Plaistow Hospital, London, E., and ask to be informed whether there are any fever hospitals in the required neighbourhood which give two years' regular training, as in many fever hospitals the course is very unsatisfactory. We need hardly add that fever nursing, if followed by general training, opens up a very lucrative career. During the waiting period your daughter could study massage, which can be learned at any age and is useful in every branch of nursing.

### Permanent Cases.

Nurse W.—We have noted your address, but, as things are, we are not able to help you. It is unsatisfactory that capable nurses should be unemployed, while members of the public find a difficulty in obtaining efficient permanent nurses.

### Nurse Secretary.

B. M.—You must remember that private nursing is not a very good training for the post of secretary to a doctor in his practice which you desire to obtain. A knowledge of typing and of shorthand are indispensable, as it is usual for busy practitioners to dictate their letters. Also business training of a general character would be required. Unless you could produce evidence of secretarial qualifications such as we have indicated you would have no chance of securing this kind of post.

### Homes Wanted.

### For Lady with Partial Paralysis.

E. H .B. (Yorkshire) (118a).—Thanks for second letter. As you can pay 25s. a week we are hopeful that some of the Homes on our List will write and offer to receive your aunt. We note that "in or near London" would be preferred, as you would like to visit her. This is a pity, as naturally London nursing homes are more expensive, but we do not despair. I am sending on a letter from a home in Yorkshire as it might be convenient to place her there for a while before bringing her south.

### Sufferer with Spinal Complaint.

F. C. M. (Tyrone) (119a).—We are quite pleased to make your want known, and will forward letters to you from homes willing to receive you and give you the kind care and treatment necessary. If you study The Hospital every week you will soon see how we manage to answer letters without replying by post. Turn over the back (blue) cover and you will find the "coupon" at the foot of the page. Please write again and tell us how much you can afford to pay.

### Miscellaneous.

### The Superfluous Hair Problem.

W. P.—Electrolysis, skilfully carried out, is undoubtedly a satisfactory method of removing superfluous hair, but in the hands of clumsy operators it is likely to produce scarring. Unfortunately electrolysis is an exceedingly expensive process, and it is also rather painful. From twenty to thirty hairs only can be removed at a sitting, and at least fifty sittings are necessary to remove an extensive growth, the charges varying with the skill of the operator from 10s. 6d. to £2 2s. per sitting. Why not try a razor? If that is objected to a fine sharp pair of scissors used regularly every day is quite effective.

### The Editor's Letter-Box.

### Women Pharmacists and Hospital Dispensing.

*To the Editor of* The Hospital.

*To the Editor of* The Hospital.

### Appointments.

The following appointments at the Manchester Royal Infirmary have been confirmed :—G. E. Sawdon, M.B., Ch.B. (Vict.), and J. C. Jefferson, M.B., Ch.B. (Vict), to be senior house surgeons; C. F. White, M.B., Ch.B. (Vict.), and P. H. Midgley, M.B., Ch.B. (Vict.), to be junior house surgeons; A. G. Bryce, M.B., Ch.B. (Vict.), and A. H. Macklin, M.B., Ch.B. (Vict.), to be house physicians.

The Executive Committee of the King Edward VII. Welsh National Memorial has confirmed the following appointments as tuberculosis physicians to act under the medical director, Dr. Marcus Paterson :—Dr. D. A. Powell, Charing Cross Hospital; Dr. Eric H. Rhys, Harries Park Hospital for Children, Lewisham; Dr. Alfred Harris, Municipal Sanatorium, Shirley; and Miss Madeleine Stuart Baker, M.D., Clifton.

